# Decreasing Door-to-Door Times for Infliximab Infusions in a Children's Hospital Observation Unit

**DOI:** 10.1097/pq9.0000000000000131

**Published:** 2019-01-21

**Authors:** Kelly C. Sandberg, Janet N. Lucien, Denise Stoll, Erica Yanney, Adam Mezoff

**Affiliations:** From the * Department of Gastroenterology, Dayton Children’s Hospital, Dayton, Ohio; ‡ Department of Pediatrics, Wright State University Pediatrics Residency Program, Dayton, Ohio; † Department of Pediatrics, Wright State University, Dayton, Ohio.

## Abstract

**Introduction:**

Children with inflammatory bowel disease (IBD) often require infliximab infusions to manage their disease. Infusions administered in the hospital setting require the patient and their families to devote many hours away from home. Changing to a rapid infusion protocol has been shown in the literature to be safe and has the potential to decrease time spent in the hospital receiving infusions.

**Methods:**

We describe stepwise changes made over a 4-month period to improve infliximab infusion efficiency and lessen the time spent in the hospital by IBD patients and their families. These changes included the implementation of a standardized order set, defaulting to rapid infusions for eligible patients, eliminating the post-infusion observation window, and improving the pharmacy's efficiency in preparing infusion medications. We utilized several established quality improvement tools, including a smart aim, key driver diagram, plan-do-study-act cycles, and statistical process control charts to measure these interventions.

**Results:**

Within three months of starting, the average door-to-door time patients spent in the hospital decreased by 128 minutes (2 hours 8 minutes). This improvement amounts to 768 minutes (12 hours 48 minutes) per year of time returned for normal childhood activities outside of the hospital. There were no infusion reactions during the period monitored.

**Conclusions:**

Implementation of a rapid infliximab infusion protocol made an impressive impact on freed family time without sacrificing patient safety. The changes we implemented could be helpful to other centers interested in decreasing in-hospital time for patients with IBD and their families.

## Abstract 2

Supplemental Digital Content is available in the text.

## INTRODUCTION

Children with moderate-to-severe inflammatory bowel disease (IBD), including Crohn’s disease and ulcerative colitis, often require biologic medications administered via infusions to manage their disease adequately. A common and effective biologic used for this purpose is infliximab, a chimeric monoclonal antibody against tumor necrosis factor-α. Time spent receiving these infusions in the hospital setting disrupts school, extracurricular activities, time spent at home, and other aspects of normal childhood development. Hospital-based infusions also expose patients to nosocomial pathogens and potential infection. Significant nursing time can also be spent in hospital-based infusions, tying up needed hospital resources.

Because infliximab is a biologic medication, its administration carries a small risk of a severe hypersensitivity reaction including anaphylaxis.^1^ There is variation in infliximab administration practices in the United States.^2^ However, there is good evidence in the literature demonstrating that rapid infliximab infusions 60 minutes in duration are safe after a child has reached the maintenance phase of his treatment with no prior symptoms of an infusion reaction.^3–5^ Shorter infusions may be safer according to the results of a Canadian study from 2011.^6^

The goal of this quality improvement (QI) project was to implement a standard practice in a Midwestern children’s hospital in which all eligible IBD patients would receive rapid infliximab infusions with a standardized process, thereby decreasing the average time spent at the hospital by patients and their families. We also hoped to simultaneously improve patient quality of life, patient satisfaction, and the efficiency of health care delivery, though we did not formally asses these metrics. If this intervention were to be adopted broadly, it could substantially decrease the cumulative hours children with IBD and their families spend receiving infusions in hospital settings nationwide. An executive sponsor supported the project, and it was completed as part of the Intermediate Improvement Science Series, supported by Cincinnati Children’s Hospital Medical Center.

## METHODS

We undertook this project in a medium-sized free-standing children’s hospital in the Midwest. In our institution at the project’s inception, 5 physicians cared for patients with IBD). Our population of patients with IBD slowly increased throughout this project, given patients were newly diagnosed, and incorporated into our data collection. At the beginning of the project, our population consisted of approximately 140 IBD patients, with approximately 50 receiving infliximab infusions, and 28 eligible for rapid infusions (RIs) based on our protocol. These patients received infusions every 4–8 weeks, depending on clinical dictate.

**Fig. 1.**
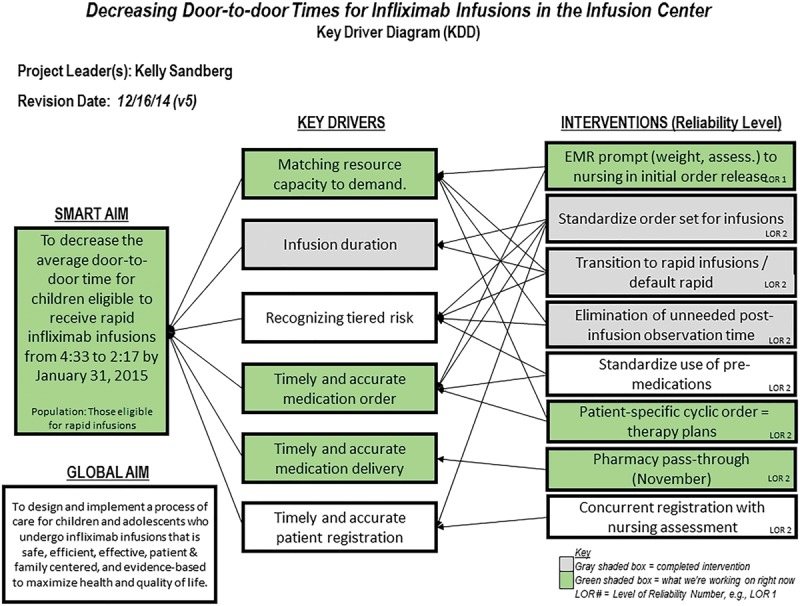
KDD, including SMART (Specific, Measurable, Actionable, Relevant, Time-bound) aim. EMR indicates electronic medical record; KDD, key driver diagram; LOR, level of reliability.

**Fig. 2.**
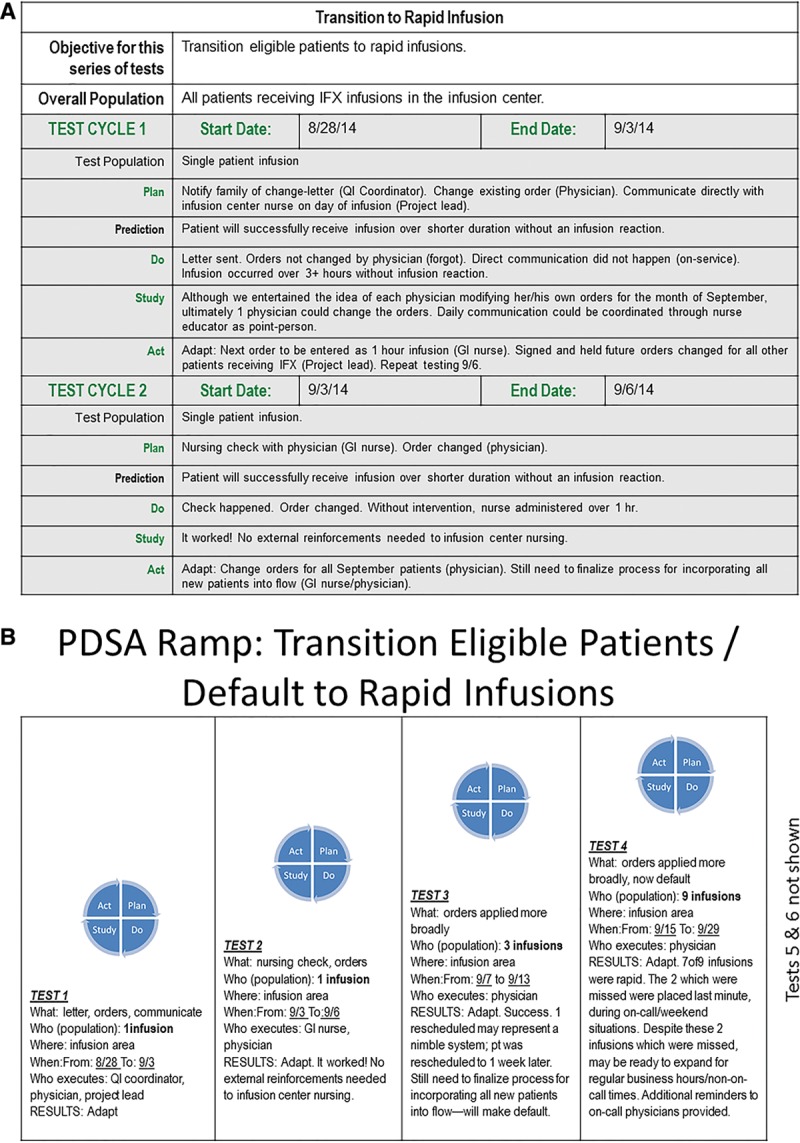
Plan-do-study-act (PDSA) cycles. A, Sample PDSA worksheet for the transition to rapid infusion. B, PDSA Ramp: transition eligible patients/default to rapid infusions. IFX, Infliximab; GI, Gastroenterology.

An interdisciplinary team consisting of 5 pediatric gastroenterologists, a gastroenterology nurse, an infusion nurse educator, an infusion nurse, a clinical pharmacist, a QI consultant, and an executive sponsor began meeting in July 2014 to address this project specifically. The QI framework was implemented to create a smart aim: to decrease the average door-to-door time for eligible children to receive rapid infliximab infusions from 279 minutes (4 hours 39 minutes) to 137 minutes (2 hours 17 minutes) over a 6-month period (Fig. 1). We set this goal from best case scenario modeling plus a 10% buffer. We define door-to-door time as when patients are registered to when they have been discharged; both were time stamped in the electronic medical record (EMR) and manually extracted. We identified key drivers and populated corresponding interventions, presented in an interim key driver diagram, complete with SMART (Specific, Measurable, Actionable, Relevant, Time-bound) aim (Fig. 1). Plan-do-study-act cycles (PDSAs; Fig. 2) which are an iterative, problem-solving modeling tool commonly used in QI work, allowed the team to learn from early failures and successes and provided a framework for spreading successful changes.

**Fig. 3.**
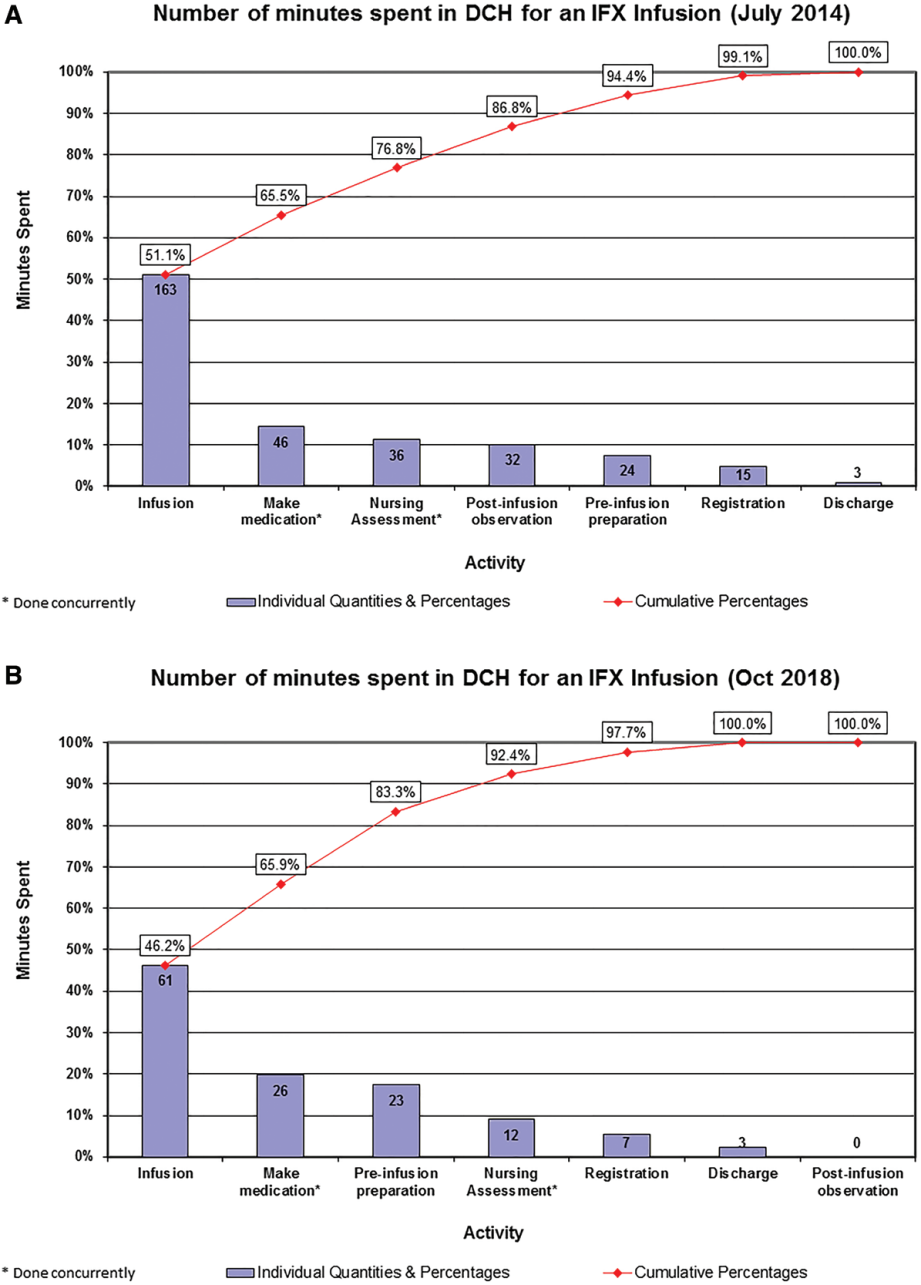
Pareto charts of door-to-door time spent by families who receive infliximab infusions at the start of the project in July 2014 (A) and in October 2018 (B). DCH, Dayton Children’s Hospital; IFX, Infliximab.

Chart reviews, confirmed with direct clinical observation, provided overall door-to-door times and interval steps of a typical patient’s infusion visit including registration, medication preparation, preinfusion nursing assessment, preinfusion preparation, infusion, postinfusion observation, and discharge (see Pareto chart, Fig. 3A). The Pareto chart guided the team toward those steps in the process (primarily the times of infusion and postinfusion observation) where we could attain the largest improvements.

Previous research has shown that RIs are safe.^3,6^ A primary intervention of this project included the transition of eligible patients to RI to decrease door-to-door times in the hospital. To be eligible, patients needed their first 3 induction infusions plus 1 maintenance infusion complete without any evidence of an infusion reaction.^3^ Written handouts were given to families in the form of letters, and discussed with families in the clinic setting and at infusions via standardized scripting before making the transition. We trained nurses and physicians to use the standardized scripting before transitioning patients. Patients, families, or medical treatment teams could opt out of RIs at any point throughout this QI project for safety or any other concerns.

We describe interventions in the Results section below. We gathered data to assess the efficiency and safety of the new process. The primary outcome measure was the door-to-door infliximab infusion times within the observation unit of the hospital. We used Microsoft PowerPoint, Excel, and Word to create a smart aim, key driver diagram, PDSA ramps, simplified failure mode effects analysis (Supplementary Digital Content 1, Figure 1, http://links.lww.com/PQ9/A66), statistical process control charts, and other tools that aided in data analysis, interpretation, and communication among target populations.

**Fig. 4.**
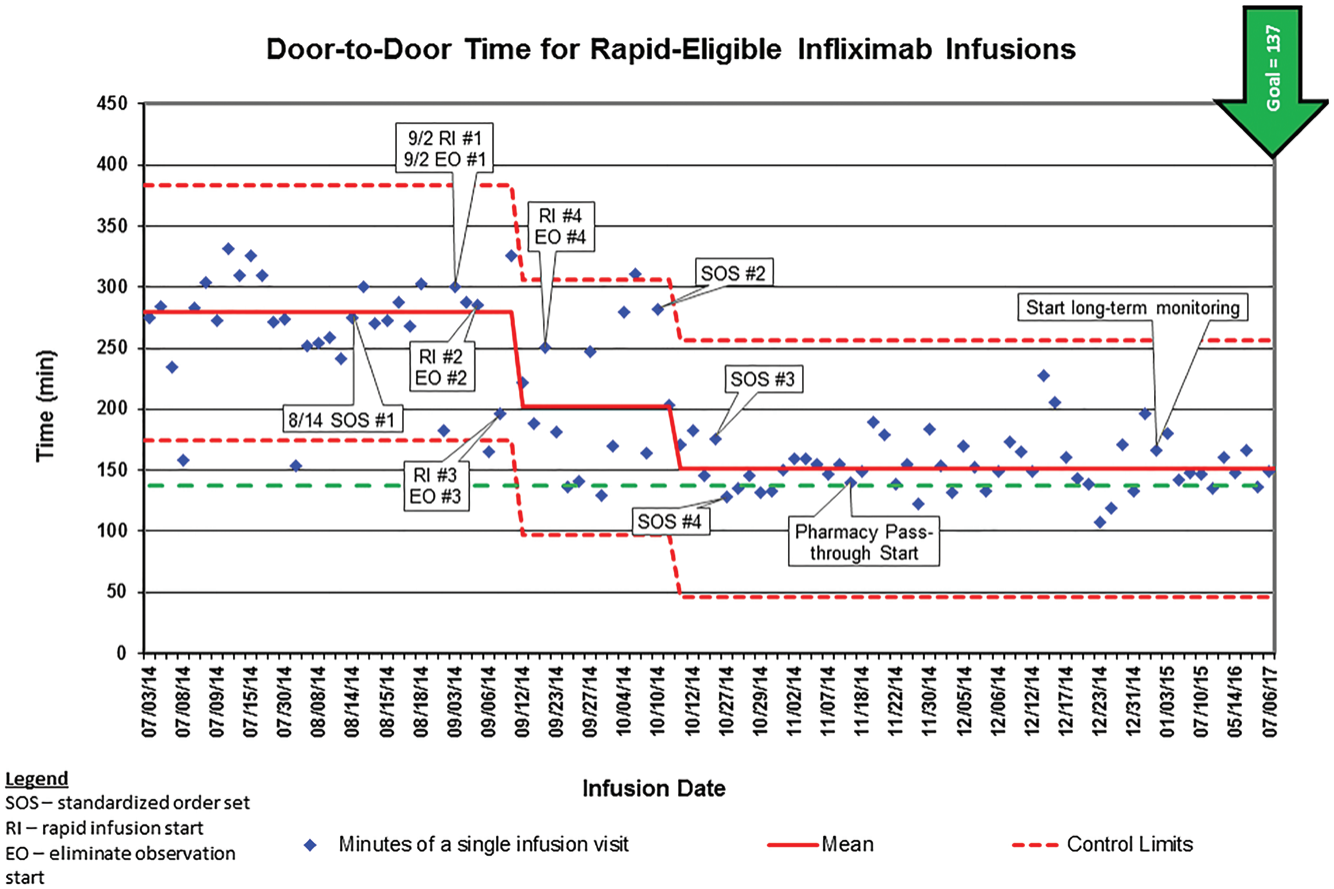
Statistical process control chart with annotations. EO indicates eliminate observation; RI, rapid infusion start; SOS, standardized order set.

The changes above were implemented initially on a small scale, and each analyzed within a PDSA framework. We made slight adjustments through several tests of change, at each iteration gaining more momentum and buy-in for broader application. We created a control chart with the data collected on door-to-door times before the project initiation (ie, for baseline data), during each test of change, and throughout the maintenance phase covering a 6-month period (Fig. 4). Each data point in Fig. 4 represents the measured minutes of a single infusion visit. After the 6-month period ended, data were collected on a less frequent basis (via a standard sampling frame) to ensure stability and to ensure that we sustained gains.

There were no anticipated ethical concerns before starting this QI initiative, nor did we uncover any throughout. There were no conflicts of interest. Our local institutional review board approved this project as being a nonresearch, QI initiative, and thus did not undergo a full committee review. We employed SQUIRE (Standards for QUality Improvement Reporting Excellence) 2.0 Guidelines in the creation of this article.

## RESULTS

Within 2 months of testing the standard order set, eliminating a postinfusion observation period (EO), and transitioning those eligible patients to RIs, the average door-to-door time decreased from a mean of 279 minutes (4 hours 39 minutes) to 202 minutes (3 hours 22 minutes), just 65 minutes longer than the initial SMART aim goal. One month later, following 2 more PDSAs, including broader application, the average door-to-door time decreased further to 151 minutes (2 hours 31 minutes), only 14 minutes longer than the smart aim goal (Fig. 4). We have sustained these improvements over multiple years.

An examination of the time obtained for each step of the new infusion process is presented in a Pareto chart as Fig. 3B. These data were taken from a sampling of 3 patients in October 2018 and demonstrates gains in virtually all steps of the process.

Using the difference in means from the control chart, we determined that these changes saved roughly 13 hours of nursing time each week. This time saved allowed for 46% more time for infusion patients. Even more importantly, these changes allowed for 128 minutes (2 hours 8 minutes) of freed family time. An additional benefit included less administration time spent in order placement through the use of a standard order set. We estimate that 120 hours were spent in planning, undertaking PDSAs, data analysis, and implementation efforts.

Concerning balancing measures, there were no adverse events, including no infusion reactions, in patients who received RIs during data collection. We received no reports of decreased quality or safety concerns from families or staff. Review of office visit documentation after RIs revealed no safety or quality concerns, including those items listed above.

### Interventions

The initial change in moving toward rapid infliximab infusions involved creating a standardized order set within the institution’s EMR. The goal of this order set was to simplify the process of placing infliximab infusion orders for the gastroenterologists on staff and making the orders more standardized regardless of provider. The typical dosing of medications (including premedications and infliximab), the infusion rate, and the multitude of nursing orders were all included. The order set made the front-end work more efficient and also supported the upcoming change toward RIs. The specific orders for each patient became more standardized and could be better anticipated by the infusion nursing staff as they prepared for the visit and cared for the patients throughout their infusions.

The next change was 2-fold: transitioning all eligible patients to RIs and eliminating the 30-minute postinfusion EO. The RI meant cutting the actual infusion time in half from 120–60 minutes. This change, plus the EO, meant 90 minutes of the door-to-door time would be reduced via this change alone. We had no infusion reactions in the preceding 2 years before the start of this QI project.

Four months from the start of the project, an additional change was made within the institution’s pharmacy to improve the efficiency of infusion preparation and further reduce the average patient door-to-door time. The pharmacy built a physical pass-through window in the wall adjacent to the infusion medication preparation area. Before this change, pharmacy technicians would have to don and doff personal protective equipment, sometimes repeatedly, during the preparation of an infliximab infusion if items needed were forgotten outside that room. After this change, other pharmacy staff could easily hand a needed item through the pass-through window to the technician preparing the infusion, thus making the entire process more efficient.

## DISCUSSION

This study showed a statistically significant decrease in average door-to-door times for patients receiving infliximab infusions in a children’s hospital observation unit. The initial 90-minute decrease was expected given the shorter infusion time and omitting the postinfusion EO. There were an additional 38 minutes of time saved thanks to improved efficiency and familiarity with the new protocol. The 2 hours 8 minutes of freed time could be returned to the patients and their families. For a patient who receives 6 infliximab infusions per year, a total time savings of 768 minutes (12 hours 48 minutes) per year could be amassed. Children with chronic disease spend more time in healthcare settings (emergency department, primary care, specialty clinics, and hospitals), and their care represents a substantial financial burden, than compared with those without a chronic disease.^7–11^ This reality impacts home life, school life, developmental aspects, and social growth. By returning this time to patients and their families, we give back a part of childhood stolen by IBD. Further steps toward a home-based or external-based infusions would further improve quality of life, and efforts are currently underway across the country to ensure this is done safely.^12^

As with most changes, there is often resistance at the start. We found that reminding the physicians to utilize the new order set, familiarizing nursing staff with changes in their orders, and getting the pharmacy involved in more efficient infusion preparation were all challenges initially. The culture change toward default RIs was surprisingly quick, however. Even with standardized scripting and adequate forewarning for patients’ families, there was still some hesitation, given concern for infusion reactions. The standardized scripting was thought to help prevent unintended variation in the initiatives, and improve communication between families and care teams.

This QI effort has several important limitations. First, we collected multiple data by hand. All attempts were made to ensure data accuracy, but there remains a possibility of human error. Second, we were reliant on our EMR for the timing of entries. Multiple reasons could have delayed a patient before their first official entry into our system, and evaded capture in our recording of data. Third, this was a project at a single center, which may compromise external validity. Smaller institutions tend to have less representation in the medical literature, however, and we felt the lessons learned here could be generalizable to other smaller institutions and also applicable to larger pediatric facilities.

We want to emphasize that this project did not reveal any patient safety concerns after adopting a RI protocol. A study at Stanford in 2017 yielded no safety concerns over a 2-year period of rapid infliximab infusions.^13^ Implementation of rapid infliximab infusion protocols nationwide could result in a considerable amount of freed time for IBD patients. Our QI work at a smaller free-standing children’s hospital suggests rapid improvement is possible with small sustained efforts. Our return on investment is roughly 1 hour invested for 1 hour returned for every 10 patients. In our eligible population for RIs, this equates to a 7-fold return of time for every hour of medical team investment.

Implementing a rapid infliximab infusion protocol at our institution was associated with decreased average door-to-door times for patients with IBD and their families, thus freeing up patient time for activities outside the hospital and improving the efficiency of health care delivery. We continue to look for ways to improve the quality of life for children with chronic disease.

## ACKNOWLEDGMENTS

The authors thank Anthony Fout, RN, who assisted with the development of the order set; Stephanie Wooten, LPN, who assisted with the changes in scheduling of infusions; Nancy Severt, RPh, the director of the pharmacy, who oversaw the pass-through expansion; and Brenda Lee, the quality improvement coach from Intermediate Improvement Science Series.

## DISCLOSURE

The authors have no financial interest to declare in relation to the content of this article.

## Supplementary Material
