# Supplementary figures and images for: Decreasing Door-to-Door Times for Infliximab Infusions in a Children's Hospital Observation Unit
Source: Pediatr Qual Saf. 2019 Jan 21;4(1):e131. doi: 10.1097/pq9.0000000000000131 (PMC6426496; doi:10.1097/pq9.0000000000000131)

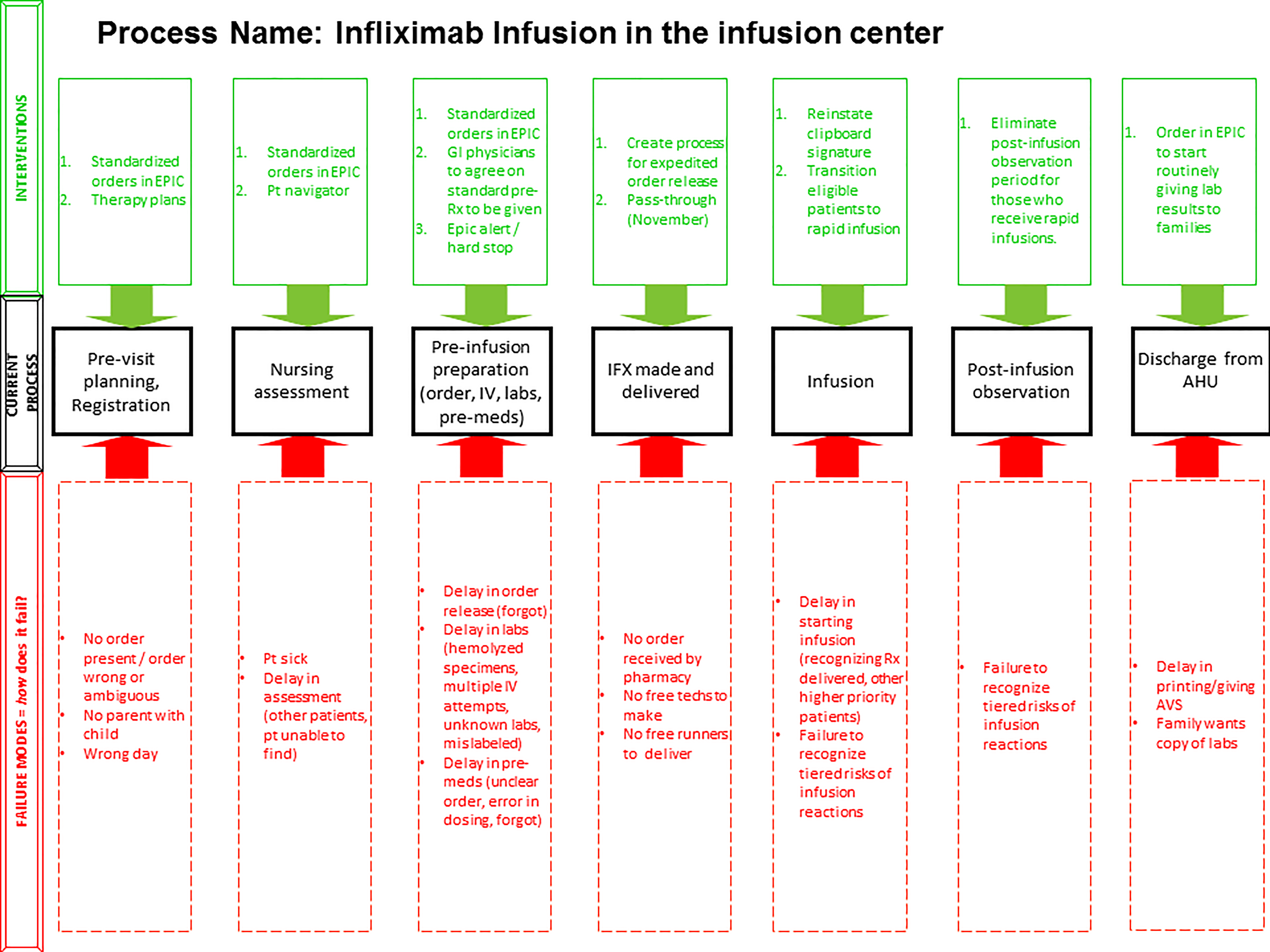

Supplement: Supplementary file 1 [file pqs-4-e131-s001.tif]
